# Chemical Characterization of Honey and Its Effect (Alone as well as with Synthesized Silver Nanoparticles) on Microbial Pathogens’ and Human Cancer Cell Lines’ Growth

**DOI:** 10.3390/nu15030684

**Published:** 2023-01-29

**Authors:** Hamed A. Ghramh, Sulaiman A. Alrumman, Irfan Ahmad, Abul Kalam, Serag Eldin I. Elbehairi, Abdulkhaleg M. Alfaify, Mohammed Elimam Ahamed Mohammed, Abdullah G. Al-Sehemi, Mohammad Alfaifi, Badria M. Al-Shehri, Rahaf Mohammed Hussein Alshareef, Wed Mohammed Ali ALaerjani, Khalid Ali Khan

**Affiliations:** 1 Biology Department, Faculty of Science, King Khalid University, P.O. Box 9004, Abha 61413, Saudi Arabia; 2 Research Center for Advanced Materials Science (RCAMS), King Khalid University, P.O. Box 9004, Abha 61413, Saudi Arabia; 3 Unit of Bee Research and Honey Production, Faculty of Science, King Khalid University, P.O. Box 9004, Abha 61413, Saudi Arabia; 4 Department of Clinical Laboratory Sciences, College of Applied Medical Sciences, King Khalid University, P.O. Box 9004, Abha 61413, Saudi Arabia; 5 Department of Chemistry, Faculty of Science, King Khalid University, P.O. Box 9004, Abha 61413, Saudi Arabia; 6 Cell Culture Laboratory, Egyptian Organization for Biological Products and Vaccines, VACSERA Holding Company, Giza 22311, Egypt; 7 Applied College, King Khalid University, P.O. Box 9004, Abha 61413, Saudi Arabia

**Keywords:** biogenic synthesis, silver nanoparticles, molecular spectroscopy, antimicrobial, anticancer

## Abstract

The antibacterial, anticancer, and wound-healing effects of honey can vary according to the type, geographical region, honey bee species, and source of the flowers. Nanotechnology is an innovative and emerging field of science with an enormous potential role in medical, cosmetics, and industrial usages globally. Metal nanoparticles that derived from silver and range between 1 nm and 100 nm in size are called silver nanoparticles (AgNPs). Much advanced research AgNPs has been conducted due to their potential antibacterial and anticancer activity, chemical stability, and ease of synthesis. The purpose of the present study was to explore the physicochemical properties of honey and the potential to use forest honey to synthesize AgNPs as well as to appraise the nanoparticles’ antimicrobial and anticancer effects. Here, we used three different percentages of forest honey (20%, 40%, and 80%) as biogenic mediators to synthesize AgNPs at room temperature. The development of AgNPs was confirmed by color change (to the naked eye) and ultraviolet-visible spectroscopy studies, respectively. The absorbance peak obtained between 464 to 4720 nm validated both the surface plasmon resonance (SPR) band and the formation of AgNPs. Regarding the sugar profile, the contents of maltose and glucose were lower than the content of fructose. In addition, the results showed that the SPR band of AgNPs increased as the percentage of forest honey increased due to the elevation of the concentration of the bio-reducing agent. A bacterial growth kinetic assay indicated the strong antibacterial efficacy of honey with silver nanoparticles against each tested bacterial strain. Honey with nanotherapy was the most effective against hepatocellular carcinoma (HepG2) and colon cancer (HCT 116) cells, with IC_50_s of 23.9 and 27.4 µg/mL, respectively, while being less effective against breast adenocarcinoma cells (MCF-7), with an IC_50_ of 32.5 µg/mL.

## 1. Introduction

Honey is a pleasant, natural, and sweet material made by honey bees from nectar and honeydew collected from flowers [1]. It is extracted from various botanical sources; has diverse chemical compositions, physical properties, and sensory features; and can be identified as a link between its attributes and its geographical origin, with beekeepers benefiting financially [2]. It is referred to as “the drink of the gods,” and is sweet to taste. In human diet, sweet nourishments have always been remained a significant part since ancient times. A temptation and enjoyment of sweet tastes is present in human beings at birth, and a fondness for sweetened flavours over plain tastes is usual through the growing period [3]. Honey, being sweet in taste has medicinal values and contains a significant amount of antioxidants, amino acids, and carbohydrates [4,5].

Honey also comprises some vitamins, minerals, enzymes, and phenolic compounds, which enable it to be a good antioxidant, boost immunity, and aid in food digestion and absorption. Despite honey’s high carbohydrate content, the glycaemic index of honey is very low, making it a good way for diabetics to stay on track with their diet [6]. Honey has been shown to have potent healing properties that combat a variety of chronic diseases and conditions [7], including cardiovascular disorders, gastrointestinal disorders [8], pulmonary disorders [9], hypertension, diabetes [10], autophagy dysfunction, and fungal and bacterial infections, which were reported in several studies. It may also be beneficial for patients with Coronavirus disease (COVID-19) [11]. Several studies have related honey’s basic physicochemical features to its botanical origin [12]. It is high in carbohydrates (80–85%) and energy, and honey’s sugars, similar to those of many fruits, are easily absorbed [13]. Most honey is made up of sugars (40% fructose, 35% glucose, and 5% sucrose) and water (20%) [14,15,16]. Enzymes, amino acids, protein, vitamins, minerals, organic acids, ash, and phenol compounds are among the more than 180 components found in honey [17], and it has some enzyme activity, hydroxymethylfurfural (HMF), and amino acids. These contribute to a vital aspect of the quality of honey [18], while the color, acidity, ash, sugar profile, and proline levels of honey are all affected by the flower source [19].

To suppress related bacteria, new and better antimicrobial treatments are continually being researched [20]. Nanotechnology is a cutting-edge technology with numerous applications in biotechnology, engineering, architecture, food security, food technologies, medical sciences, and other important sectors [21]. As a result of the introduction of nanotechnology, numerous related industries have seen significant growth in recent years [22]. Top-down and bottom-up approaches are the two main types used for creating nanoparticles [23]. Nanotechnology is currently thought to be a reliable medicinal tool and involves the creation of various nano-materials [24,25]. Magnesium, copper, gold, and silver have all been used in the construction of such structures [26]. Each metal has optical properties, which can be used to make metallic nanoparticles [27], and there are a variety of physical and chemical processes for its creation [28]. However, plants, on the other hand, are now frequently employed in the green synthesis process [29] and the process of making honey [30] because of their purity, low toxicity, and environmental friendliness. Notably, pH, osmolarity, hydrogen peroxide, and phytochemical content can increase the ability of honey to kill bacteria [31]. Some of the investigations have looked into the immunomodulatory effects of honey [32], and there are also a number of other factors, including the honey type, its geographic region and distribution, and plant characteristics, which can enhance the antibacterial activity of honey [33]. Moreover, cancer is treated using radiotherapy and chemotherapy, but these treatments are expensive and toxic to the normal cells in our bodies [34]. As a result, recent research has mostly concentrated on finding natural products, such as honey, that can help treat cancer. It has been demonstrated that honey can enhance anticancer effects [34,35].

Silver nanoparticles (AgNPs) are currently the subject of much new research since they have many biological applications [36,37,38]. AgNPs are promising antibacterial agents for wound and burn treatment [39]. Because of their demonstrated capabilities as antibacterial, antiviral, and antifungal agents, AgNPs have been used in many biotechnology fields [40,41]. In addition, the concentration of honey employed in manufacturing AgNPs is directly correlated with the AgNPs’ antifungal action [42]. Silver nitrate is reduced by the use of honey and also acts as a stabilizing mediator for AgNPs created as part of an environmentally friendly process for fabricating silver nanoparticles (AgNPs) at low temperatures [43]. Traditional medicine uses Somra (Acacia) and *Calotropis procera* honey. The benefits of mixing Somra honey (20%) and a *C. Procera* extract—for which water has been used to extract important components from the leaves—have been investigated, while the honey/leaf extract has been used to create AgNPs independently, with positive results [44]. Utilizing Indonesian wild honey as a mediator, AgNPs have been effectively synthesized using a simple and environmentally acceptable ‘green synthesis’ process [45]. Sidr honey and its synthesized AgNPs have been studied for their antibacterial properties and their potential to be employed as antimicrobial agents [30].

The main purpose of the present research was to evaluate honey’s physicochemical properties and its interaction with silver nanoparticles in terms of inhibiting microbial and human cancer cell line growth. 

## 2. Materials and Methods

### 2.1. Chemicals and Reagents

Each chemical and reagent used was of superior quality and according to the high-performance liquid chromatography (HPLC) standards. Sucrose, fructose, and maltose were purchased from the Indian based companies named Central Drug House (P) Ltd. New Delhi, India; Loba Chemie Pvt. Ltd. Mumbai, India; and Techno Pharmchem, New Delhi, India respectively. While, glucose was bought from th Chem-LabNV, Zedelgem, Belgium. However, 5-(hydroxymethyl) furfural, Mueller Hinton Broth, Sulforhodamine B sodium salt solution, and AgNO_3_ were purchased from the USA based company Sigma-Aldrich (St. Louis, MO, USA). HPLC standard grades water and acetonitrile were bought from Indian based company Chemie Pvt. Ltd. Mumbai, India. While, Roswell Park Memorial Institute (RPMI) 1640 medium and Penicillin-Streptomycin (10,000 U/mL ) were purchased from Thermo Fisher Scientific Inc. (Waltham, MA, USA). Fetal bovine serum (FBS), Trichloroacetic acid (TCA), acetic acid, and tris base were purchased from the Merck, Darmstadt, Germany.

### 2.2. Physicochemical Analysis of Honey 

The pH and moisture of honey were measured and documented according to the International Honey Commission’s (2009) harmonized methods [46]. Briefly, pH was measured in 13% (*w*/*v*) honey after calibrating the pH meter with buffer solutions of pH 4 and pH 7. The moisture content was determined using a refractometer by applying one drop of honey on the prism of the refractometer. 

#### Sugar Analysis Using a High-Performance Liquid Chromatography-Refractive Index Detector (HPLC-RID)

An electric balance (Shimadzu, Kyoto, Japan) (100 mL) was used to measure 1.25 g of each sample in a glass beaker. Then, a 25 mL total volume was made by filling the glass beaker with high-performance liquid chromatography (HPLC)-grade water. A vortex was used to dissolve the honey and filter it through 0.22 mm syringe filters (Labgeräte GmbH, Iso-lab). An HPLC system (Agilent 1260 Infinity II) with a pump (1260 Quat Pump VL) and a vial sampler (1260 vial sampler) was used to measure the contents of glucose, maltose, fructose, and sucrose [47], with the carbohydrate ZOz column (4.6 × 150 mm, 5 m), with an isocratic mobile phase of acetonitrile (water with a ratio of 75:25 was used, *v/v*; the flow rate was kept at 1.0 mL/min, and a refractive index detector (1260 RID) was used). At 35 °C, a 10-liter sample was injected and examined. Fructose and glucose standard curves were primed using HPLC-grade water at values ranging from 0.0625 percent to 2.00 percent (*w*/*v*), while sucrose and maltose standard curves were primed at 0.03125 percent to 1.00 percent (*w*/*v*). The Chem Station Edition. Rev. C.01.10 (201) software was installed. All of the equipment and software were purchased from Agilent Technologies, Santa Clara, CA, USA. The retention time of the standard was synchronized with the chromatographic peaks that corresponded to each sugar. To determine the association between the peak area and concentration, a calibration curve was created using successive dilutions of standards and fitted using linear regression analysis. The sugar content was given as a percentage [48].

### 2.3. Synthesis of Nanoparticles

The honey sample was obtained in the Saudi Arabian city of Abha. Sigma Aldrich’s AgNO_3_ was purchased and utilized exactly as directed. To make the solutions, double-distilled water was used.

#### 2.3.1. Green Synthesis of AgNPs 

In this study, 2, 4, and 8 mL of honey were dissolved in 8, 6, and 2 mL of double-distilled water to yield 20, 40, and 80% forest honey solutions, respectively. Different percentages of the honey solutions (20%, 40%, and 80%) and AgNO_3_ (0.01 M) solutions were combined in simple reactions, and the mixtures were placed in an ultrasonic bath for 30 min and maintained at room temperature. The color of the mixture solution changed to brown, which indicated the formation of AgNPs. The solutions were denoted by the abbreviations AgNP-1, AgNP-2, and AgNP-3. The materials were stored in a refrigerator for future studies.

#### 2.3.2. Characterisation

The characterization and optical-sensing application of the AgNPs were obtained using an ultra-violet-visible, double-beam spectrophotometer (PG Instrument). Scanning electron microscopy (JSM 6360 SEM/EDX) was used to scan the geometry and structure of the produced AgNPs.

### 2.4. Well-Diffusion Method for Antibacterial Susceptibility Assay

For evaluating the studied bacteria’s vulnerability to honey alone (20%, 40%, and 80%) and honey with AgNPs (20%, 40%, and 80%), the growth of the bacterial strains until the logarithmic phase, i.e., O.D._610_ of 0.4–0.6, took place in a Mueller Hinton (MH) broth. After that, further dilution of the tested strains of bacteria was carried out in an MH broth until a theoretic O.D._610_ of 0.01 was reached. In order to determine the antibacterial effectiveness of honey and honey with AgNPs, the agar well-diffusion method was used [49]. A sterile syringe cap was used to create wells with a 6 mm diameter in nutritional agar, and a sterile cotton swab with a diluted culture was used for the production of a lawn culture on the agar. After that, 20 μL volumes of honey and honey with silver nanoparticles were placed in a Petri dish with triplicate wells and incubated aerobically at 37 °C for 24 h. The diameter of the clear zone, where the growth of bacteria had been inhibited, was calculated in millimeters, and the well diameter was calculated in millimeters.

### 2.5. Growth Kinetic Assay

A logarithm of comparable size to the population of bacteria at various time intervals was used to construct a bacterial growth curve that illustrated the viability of the bacteria. The bacterial growth curve was employed to estimate the efficacy of honey and honey with AgNPs as antibacterial agents. Throughout 16 h of bacterial cultivation, the optical density (O.D._600_) was taken to establish the growth curve of the bacteria. To explore the efficacy of the honey and honey with AgNPs in inhibiting the tested bacterial strains, 20 μL of volumes of honey and honey with AgNPs were combined with 180 μL of the bacterial culture (O.D._600_ of 0.01). To serve as a control, a bacterial culture well with no treatment was used. This assay was performed in 96-well plates, which were incubated aerobically for 16 h at a temperature of 37 °C, and the optical density was recorded every 2 h at intervals of 600 nm using a plate reader (FLUOstar Omega, BMG Labtech, Allmendgrun, Ortenberg, Germany). Every set of experiments was repeated thrice to determine average values. The average optical density was plotted against time. 

### 2.6. Anticancer Properties of Honey

#### 2.6.1. Cell Culture

American Type Culture Collection (ATCC) human mammary gland, breast adenocarcinoma (MCF-7), human colon adenocarcinoma (HCT 116), and hepatocellular carcinoma (HePG2) cell lines were collected and grown in RPMI1640 medium, Gibco, USA. FBS (fetal bovine serum) (10%) and 100 units/mL of PS (penicillin/streptomycin) were added to the cultural mix. The cells were incubated at a temperature of 37 °C in a humidified environment containing 5% CO_2_ [50].

#### 2.6.2. Cell Viability Assay

Additionally, 96-well plates were seeded with MCF-7, HeLa, and HePG2 cells, at approximately 105/well. Following 72 h exposure to honey and honey with AgNPs, the medium was changed to 150 L of 10% trichloroacetic acid (TCA) from Merck for 1 h at 4 °C; then, the cultures were washed five times with distilled water. Afterward, a 70 μL SRB solution (0.4% *w*/*v*) (Sigma Aldrich, St. Louis, MO, USA) was added for 10 min at room temperature in a dark location. The cells were washed three times with 1% acetic acid (Merck) and left overnight to air dry. The protein-bound SRB stain was dissolved by adding 150 μL of 10 mM Tris Base (Merck), and the O.D. was measured at 540 nm using a FluoStar Omega microplate reader (BMG Labtec, Ortenberg, Germany) [51].

## 3. Results

### 3.1. Physiochemical Properties of Honey

Our results relating to the sugar profile of honey are shown in Table 1. Concerning the sugar profile, the fructose content was the highest (38.80 ± 0.39), followed by the contents of glucose (24.31 ± 0.44) and maltose (1.45 ± 0.24), while the sucrose content was the lowest (0.76 ± 0.03). 

**Figure 1:**
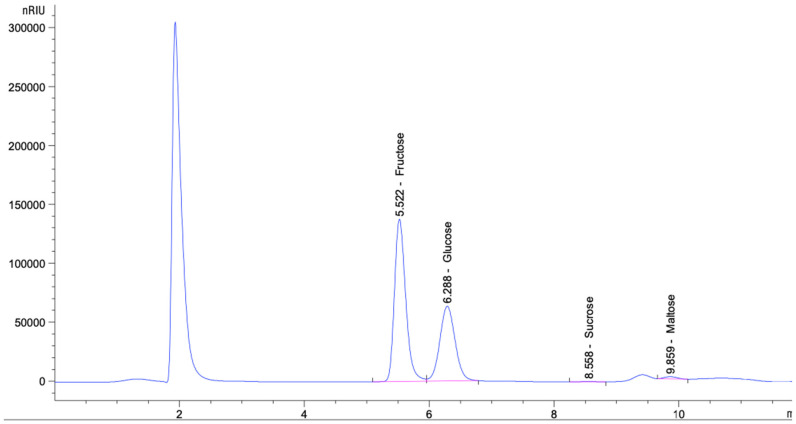
Sugar contents in honey.

The identification and quantification of the main sugar levels in honey were determined using an HPLC analytical approach in this study. At retention durations of 6.080, 7.015, 10.004, and 12.214 min, fructose, glucose, sucrose, and maltose peaks could be identified (Figure 1). Fructose, glucose, sucrose, and maltose peaks were observed at retention durations of 6.077, 7.024, 10.098, and 12.085 min, respectively (Figure 1).

### 3.2. UV—Vis Spectra of Forest Honey 

**Figure 2:**
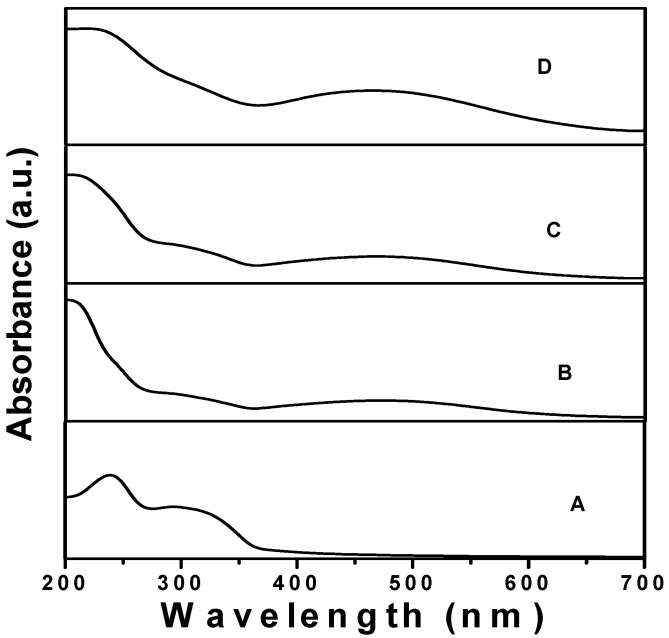
UV–vis spectra of forest honey (**A**) and AgNPs using 20% (**B**), 40% (**C**), and 80% honey (**D**).

The absorption spectra of the AgNPs synthesized using different percentages (20, 40, and 60%) of forest honey are presented in Figure 2. It is clear from Figure 1 that the AgNPs synthesized using 20%, 40%, and 80% honey showed absorption bands at 472 nm and 468 nm, whereas the absorption band was obtained at 464 nm for the AgNPs synthesized using 80% honey. In addition, a blue shift in the SPR band was observed when the concentration of honey was increased from 20 to 80%. The observed results also indicated that a different size of AgNPs was obtained using a different percentage of honey. 

### 3.3. Scanning Electron Microscopy (SEM)

**Figure 3:**
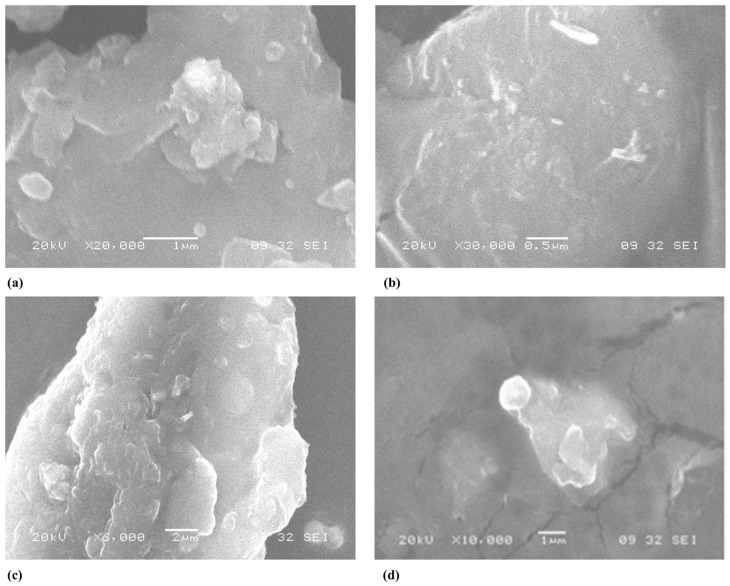
Scanning electron microscope (SEM) images of forest honey (**a**) and AgNPs synthesized using 20% (**b**), 40% (**c**), and 80% forest honey (**d**).

The SEM micrographs of the pure forest honey and AgNPs synthesized using different percentages (20, 40, and 80%) of forest honey are shown in Figure 3. It is clear from the images that the AgNPs had a round surface and a spherical shape with the 20% and 80% concentrations of forest honey, but they were rice-shaped with 40% forest honey. It has been stated that nanoparticles aggregate excessively in solution. 

### 3.4. Antibacterial Efficacy of the Synthesised Compounds

**Figure 4:**
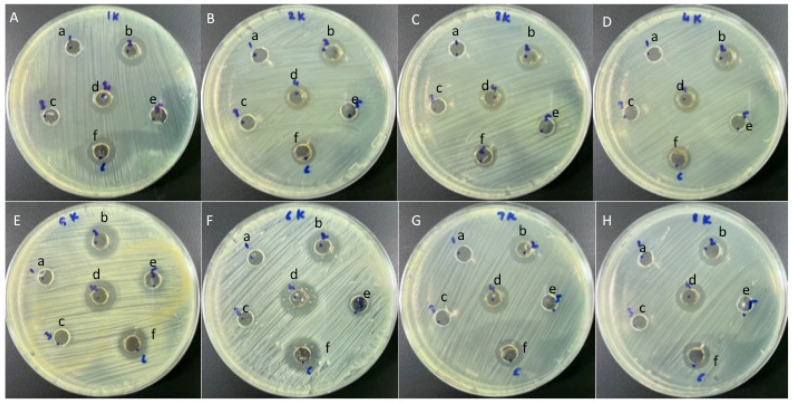
(**A**–**H**). Antibacterial properties are exhibited by the zones of inhibition produced by honey and honey with AgNPs (a = 20% honey, b = 20% honey with AgNPs, c = 40% honey, d = 40% honey with AgNPs, e = 80% honey, and f = 80% honey with AgNPs). (**A**) *Pseudomonas aeruginosa*; (**B**) *Escherichia coli*; (**C**) *Klebsiella pneumoniae*; (**D**) *Salmonella*; (**E**) *Staphylococcus aureus*; (**F**) *Staphylococcus saprophyticus*; (**G**) *Streptococcus pyogenes*; (**H**) *Enterococcus faecalis*.

**Figure 5:**
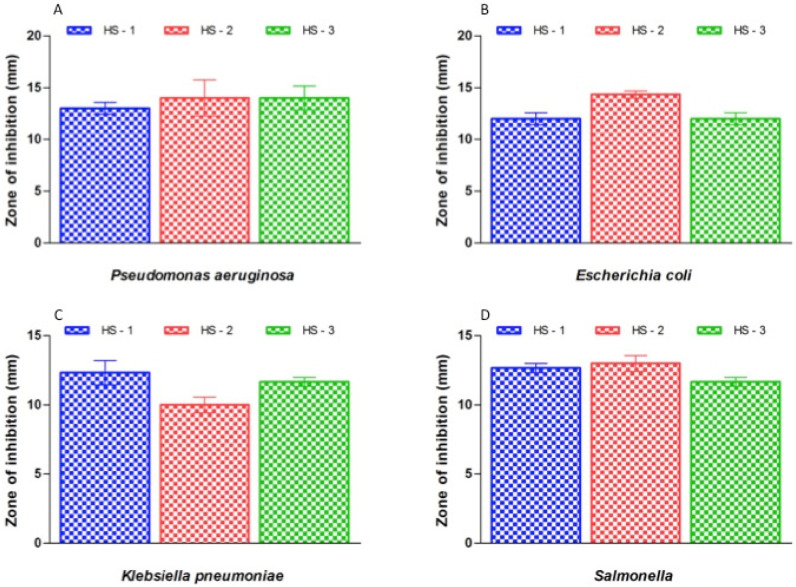

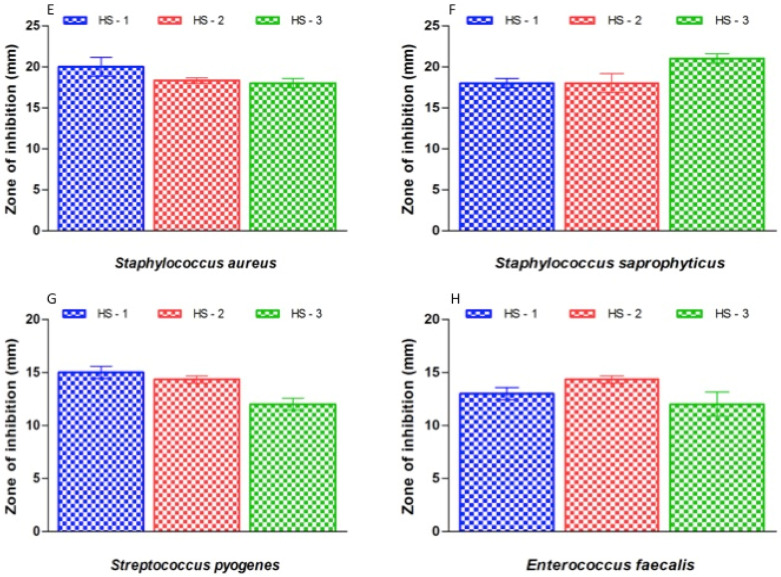
(**A**–**H**). Inhibitory efficacy of the honey with AgNPs. (HS-1: 20% honey with silver nanoparticles; HS-2: 40% honey with AgNPs; HS-3: 80% honey with AgNPs). (**A**) *Pseudomonas aeruginosa*; (**B**) *Escherichia coli*; (**C**) *Klebsiella pneumoniae*; (**D**) *Salmonella*; (**E**) *Staphylococcus aureus*; (**F**) *Staphylococcus saprophyticus*; (**G**) *Streptococcus pyogenes*; (**H**) *Enterococcus faecalis*.

Gram-negative and Gram-positive bacterial isolates were treated to test the antibacterial efficacy of honey and honey with AgNPs (Figure 4). Susceptibility studies showed that the honey with AgNPs showed antibacterial properties for all the tested bacterial types, while honey alone exhibited no effect against any of the tested bacterial strains (Figure 4). Honey with AgNPs showed the highest antibacterial activity against *S. saprophyticus* (17 to 21 mm), while the lowest antibacterial activity was observed against *K. pneumoniae* (10 to 12 mm) (Figure 4 and Figure 5).

### 3.5. Effect on Bacterial Growth 

**Figure 6:**
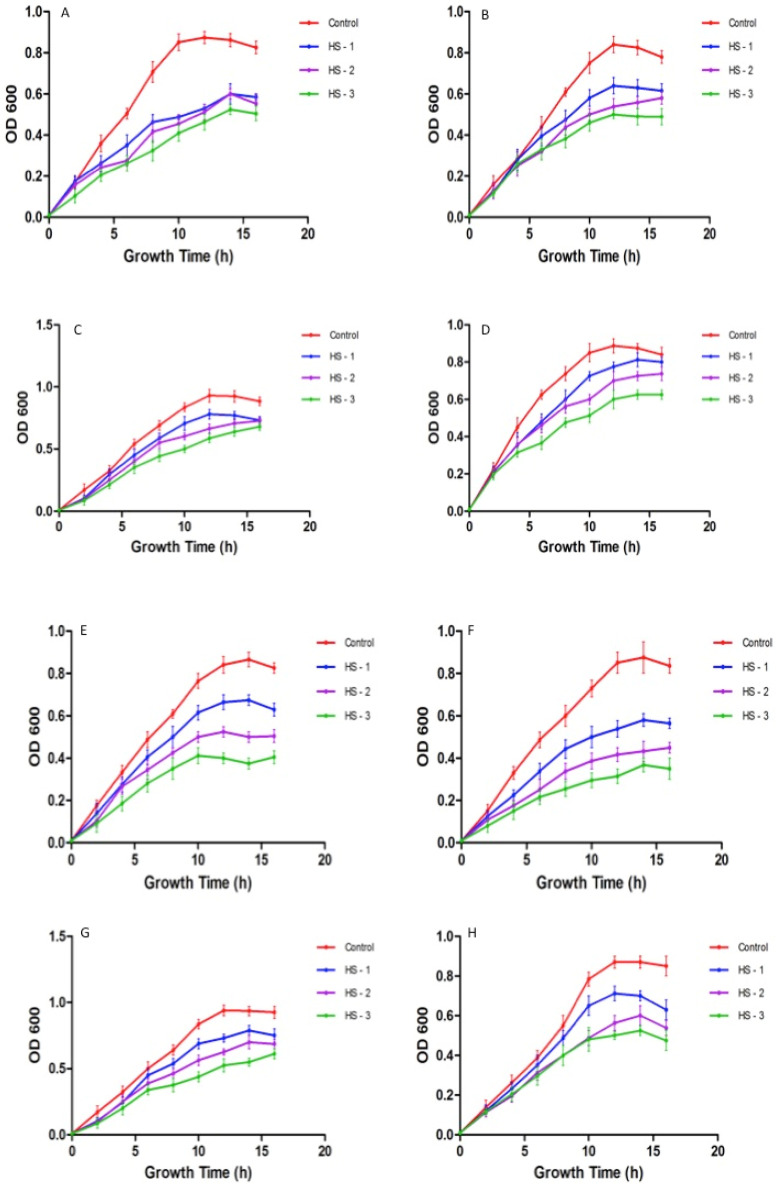
(**A**–**H**)**.** Effect of honey with silver nanoparticles on microbial growth. (HS-1: 20% honey with silver nanoparticles; HS-2: 40% honey with silver nanoparticles; HS-3: 80% honey with silver nanoparticles). (**A**) *Pseudomonas aeruginosa;* (**B**) *Escherichia coli;* (**C**) *Klebsiella pneumoniae;* (**D**) *Salmonella;* (**E**) *Staphylococcus aureus;* (**F**) *Staphylococcus saprophyticus;* (**G**) *Streptococcus pyogenes;* (**H**) *Enterococcus faecalis*. The growth cycle of untreated organisms served as the growth control. Optical density at 600 nm was measured at regular time intervals of 2 h.

The bacterial growth curve of honey with AgNPs was used to examine the efficacy of different concentrations of honey with AgNPs on bacterial growth at various time intervals. The growth progress of the bacteria was examined for 16 h (Figure 6). It is self-evident that the bacterial isolates’ growth was slowed as a result of their treatment. The efficacy of the honey containing silver nanoparticles in inhibiting each investigated bacterial strain was demonstrated upon testing the bacterial growth kinetics.

### 3.6. Anticancer Effect on Different Cell Lines

**Figure 7:**
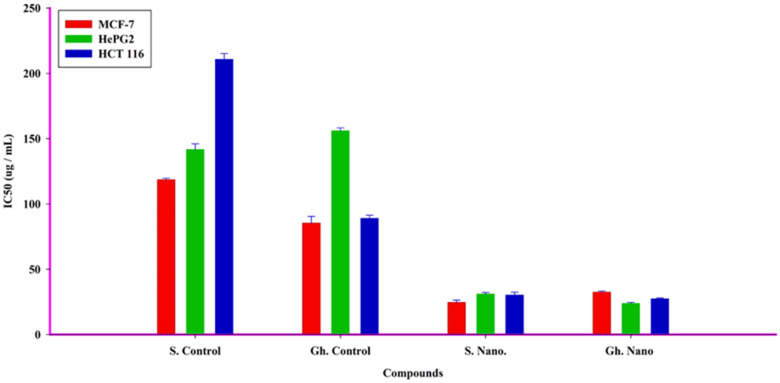
Cytotoxic effects of honey (Gh = Forest and S = Sider) and nanoparticles on different human cell lines.

**Figure 8:**
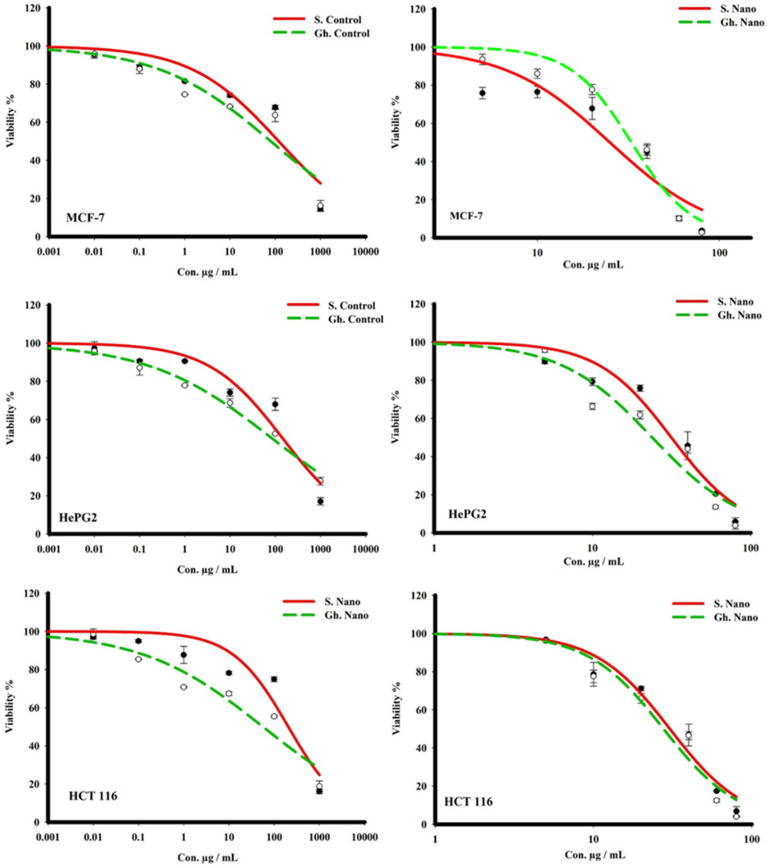
The different IC_50_s of honey (Gh = Forest and S = Sider) and honey and nanoparticles against three human tumor cell lines.

Human cancer cell growth was shown to be inhibited by treatment with honey and honey with nanoparticles. The SRB assay was conducted for MCF-7, HCT 116, and HePG2 cell viability following treatment with increasing concentrations of honey and honey with nanoparticles for 72 h; the IC_50_ values are illustrated in Table 2. Honey (Gh = Forest) with nano-therapy was the most effective against hepatocellular carcinoma (HepG2) and colon cancer (HCT 116), with IC_50_s of 23.9 and 27.4 µg/mL, respectively, while being ineffective against breast adenocarcinoma cells (MCF-7), with an IC_50_ of 32.5 µg/mL. Meanwhile, the honey (S = Sider with nano-therapy showed a promising killing effect against MCF-7 cells, with an IC_50_ of 24.6 µg/mL, compared to HepG2 and HCT 116 cells, with IC_50_s of 31.08 and 30.3 µg/mL, respectively. The S. Control, on the other hand, showed a low cytotoxic effect on the three cancer cell lines, with IC_50_s greater than 100 µg/mL, as did the Gh (Figure 7). The control showed a low effect on HePG2 cells, but the Gh did not. The control had a moderately toxic effect on MCF-7 and HCT 116, with IC_50_s of 85.5 and 89.02 µg/mL, respectively (Figure 8).

## 4. Discussion

The main purpose of this study was to evaluate and investigate the physicochemical properties of honey and its interaction with nanoparticles in inhibiting the growth and viability of bacteria and human cancer cell lines. Our study reported that the fructose content was higher than the contents of glucose and maltose. Previous studies demonstrated that the total sugar concentration of Manuka honey in New Zealand was significantly lower than that of Saudi honey (*p*-value = 0.006) and Hungarian honey (*p*-value = 0.006) [52,53]. Moreover, the Manuka honey’s moisture percentage was significantly higher than that of the Saudi and Hungarian honey (*p*-values = 0.009 and 0.015, respectively). Our findings are consistent with those of Ismail et al. [54], showing that fructose is the main sugar type present in all studied wild honey, with the highest quantity found in blossom honey (46 g/100 g).

Nanotechnology is among the most auspicious technologies in current areas of study in modern science. Our results demonstrated that different sizes of AgNPs were obtained when using different percentages of honey. In addition, the AgNPs had a round surface and spherical shape with 20 and 80% forest honey but had a rice shape with 40% forest honey. This synthesis is more practical because honey works as a stabilizing and reducing agent. However, the main challenges with honey-based nanoparticles are the necessity of defining the compounds in honey that cause metal reduction and determining which of the several available honey applications is suitable for manufacturing nanoparticles.

In this study, we explored the efficacy of different concentrations of honey with silver nanoparticles in inhibiting Gram-negative and Gram-positive bacteria. Honey with AgNPs showed positive effects against all of the tested bacterial strains, producing zones of inhibition that ranged from 10 to 21 mm. Our study observed that honey with AgNPs showed the highest antibacterial effect against Gram-positive bacterial flora. Hypothetically, the dissimilarity in the absorption band is due to the deviation of the color of AgNPs, even though the deviation of color relates to the size of the nanoparticles [55,56]. The characteristic absorption spectra obtained in the range of 472 nm to 464 nm due to surface plasmon resonance (SPR) prove that the synthesis of AgNPs at room temperature using honey is possible [57,58].

Almalki et al. [59] found similar results when they tested the nanoparticles against Gram-positive *Bacillus subtilis* and Gram-negative *Escherichia coli*. The results revealed that the chemicals tested were particularly efficient against Gram-positive bacteria. Differences in the cell wall architectures and antibacterial mechanisms of AgNPs for different cell types could explain the disparities in susceptibility to silver nanoparticles [60]. Gram-positive bacteria, for example, have a thick peptidoglycan layer, but there is a thin layer of peptidoglycan in Gram-negative bacteria; the latter also contain an outer layer made up of lipids, making them more complicated and resistant to AgNP diffusion. In the logarithmic growth phase, inhibitory effects were visible. Similarly, it was recently revealed that Gram-positive bacteria are most susceptible during the exponential growth phase [61]. Employing nanoparticles as antibacterial agents is linked to bacterial resistance. Honey combined with AgNPs could be used as an alternative antibacterial agent without the negative side effects associated with disinfectants, antibiotics, and other standard antimicrobials [62]. 

In addition, our findings demonstrated that the growth of human cancer cells was inhibited by treatment with honey and honey with nanoparticles. However, honey with nanotherapy was most effective against hepatocellular carcinoma (HepG2) and colon cancer (HCT 116) cells, whereas it was ineffective against breast adenocarcinoma cells (MCF-7). Our results are in line with the findings of Ghramh, Ibrahim, and Kilany [30], who reported that honey and honey with AgNPs had anticancer effects against HepG2 cells. Furthermore, Acacia honey showed potent anticancer activity against breast (MCF-7), colon (HCT-116), and lung (A549) cancer cell lines [63]. Another study reported similar findings in that Majra honey demonstrated anticancer effects on both the HepGe2 and Hela cell lines when combined with AgNPs; however, honey alone did not have this effect on Hela cells [64].

Our findings were restricted to biological activities because we did not investigate the underlying mechanism through which metal oxide nanoparticles created using a honey-mediated process act. Thus, further studies are required to determine the mechanism through which nanoparticles created through honey-mediated processes act in drug delivery and medical applications. 

## 5. Conclusions

In the present study, the forest honey was found to be acidic in nature and moisture content was 16.66% which is according to the international standards. The fructose content was the highest, followed by the contents of glucose, maltose, and sucrose. The synthesis AgNPs was validated by means of UV-Vis spectroscopy in 464 nm and their size was ascertained through SEM and verified to be smaller than 100 nm with diverse outlines. Furthermore, due to the presence of a high concentration of the bio-reducing agent, the SPR band of AgNPs increased as the percentage of forest honey increased. Antibacterial studies revealed that the honey with AgNPs had antibacterial properties for all tested Gram-negative and Gram-positive bacterial strains, while honey alone exhibited no effect against any of the tested bacterial strains. A bacterial growth kinetic test revealed that honey containing silver nanoparticles had good antibacterial activity against each tested bacterial strain. Honey with nanotherapy was most effective against hepatocellular carcinoma (HepG2) and colon cancer (HCT 116) cells, while it was ineffective against breast adenocarcinoma cells. Further clinical trials are required to explore the roles of various biomolecules in the biosynthesis of honey with AgNP nanoparticles and its use in medical fields. 

## Figures and Tables

**Table 1:** Analysis of physicochemical properties of honey.

Parameters	Minimum	Maximum	Mean	Std. Deviation
Fructose	38.36	39.12	38.80	0.39
Glucose	23.91	24.78	24.31	0.44
Sucrose	0.05	0.10	0.076	0.03
Maltose	1.18	1.63	1.45	0.24
pH	3.80	3.82	3.80	0.01
Moisture	16.00	17.00	16.66	0.57

**Table 2:** The IC_50_s of honey (Gh = Forest and S = Sider) and honey and nanoparticles for MCF-7, HePG2, and HCT 116 human tumor cells.

Treatments	IC_50_ (µg/mL)		
	MCF-7	HepG2	HCT 116
S. Control	118.6 ± 1.1	141.6 ± 4.5	210.9 ± 4.5
Gh. Control	85.5 ± 5.1	156.1 ± 2.3	89.02 ± 2.4
S. Nano	24.6 ± 1.8	31.08 ± 1.1	30.3 ± 0.2
Gh. Nano	32.5 ± 0.7	23.9 ± 0.8	27.4 ± 0.7

## Data Availability

Data is contained within the article.
